# Overcoming Bias: Cognitive Control Reduces Susceptibility to Framing Effects in Evaluating Musical Performance

**DOI:** 10.1038/s41598-018-24528-3

**Published:** 2018-04-18

**Authors:** Gökhan Aydogan, Nicole Flaig, Srekar N. Ravi, Edward W. Large, Samuel M. McClure, Elizabeth Hellmuth Margulis

**Affiliations:** 1 Department of Psychology, Arizona State University, Tempe, Arizona USA; 2 Department of Psychological Sciences, University of Connecticut, Storrs, Connecticut USA; 3 Department of Music, University of Arkansas, Fayetteville, Arkansas USA

## Abstract

Prior expectations can bias evaluative judgments of sensory information. We show that information about a performer’s status can bias the evaluation of musical stimuli, reflected by differential activity of the ventromedial prefrontal cortex (vmPFC). Moreover, we demonstrate that decreased susceptibility to this confirmation bias is (a) accompanied by the *recruitment* of and (b) correlated with the *white-matter structure* of the executive control network, particularly related to the dorsolateral prefrontal cortex (dlPFC). By using long-duration musical stimuli, we were able to track the initial biasing, subsequent perception, and ultimate evaluation of the stimuli, examining the full evolution of these biases over time. Our findings confirm the persistence of confirmation bias effects even when ample opportunity exists to gather information about true stimulus quality, and underline the importance of executive control in reducing bias.

## Introduction

By modulating expectations and beliefs, contextual information can alter the enjoyability of stimuli as diverse as artworks, soda, and wine^1–3^, influencing or even dominating actual sensory perception^4^. Normative theories about rational decision-making struggle to explain this phenomenon^4^, despite its potentially detrimental consequences for political, educational and economic decision-making. For instance, information about the gender, ethnicity, or background of the person assessed can distort the evaluation of identical work outputs^5–7^.

Conversely, contextual information can contribute materially to positive perceptual experiences. Aesthetic experiences sometimes depend on the prior activation of a set of beliefs that dispose a person to perceiving this way—a “preparatory set” consisting of expectations and beliefs^8^. For instance, even though listening to Joshua Bell perform a concert on the violin can cost $100 per ticket, an incognito performance by him at a subway station triggered very little interest^9^. Generally, this evidence suggests that contextual information can affect preferences and perception in both nefarious and beneficial ways.

Previous neuroimaging studies suggest that the influence of beliefs and expectations arises not merely from the sensory system, but from the particular sensitivity to contextual information of reward structures in the brain. In the realm of gustatory experience, information about price^2,3^ and brand^10^ has been shown to impact self-reported taste preferences and neural activity in the ventromedial prefrontal cortex (vmPFC) – a central component of the brain’s reward network^11,12^. Similarly, experienced olfactory pleasantness changes in response to information about a smell’s source (cheese versus sweaty socks) varies with corresponding vmPFC activation^13^. Greater vmPFC activation also occurs when paintings are labelled as having been sourced from a gallery rather than computer generated^1^.

Although the *cognitive* processes underlying framing bias have received increasing attention in recent years, there is surprisingly scarce evidence regarding the *neural* mechanisms that underlie decision-makers’ ability to reduce the influence of contextual information to form a less biased (or more objective) decision. Here, we examine whether the recruitment of cognitive control processes reduces one’s susceptibility to framing effects, akin to overcoming a prepotent response. Based on previous findings that demonstrate the importance of the reward network in expressing this behavioral bias, we argue that a stronger integration of the reward network with cognitive control related areas might be important in mitigating those behavioral biases. That is, if reward related neural processes cause framing effects, then the structural connectivity between the executive control network and areas in the reward network might also mitigate this behavioral bias. This notion is supported by findings that demonstrate the ability of the cortico-striatal circuit to connect important parts of the cognitive control network with reward structures in the cortex^14,15^.

Thus, we hypothesize that dlPFC activation — a crucial part of the executive control network^16–21^ — and its structural connectivity with reward areas correlates with a reduction in susceptibility to framing effects in perceived musical performance. To test this, participants were scanned using fMRI while they listened to pairs of piano excerpts, one of which they were told was performed by a professional, and one of which they were told was performed by a student. We examined activity during the period when they received the prime (professional vs. student), the period when they listened to the music, and the period when they made the decision about which performance they preferred, to understand the time course and mechanism by which contextual information can impact the felt value of an experience. We analyzed the neural processes and anatomical structures that led to a positive bias in favor of the performance primed as professional, and also to conditions when this bias was successfully reversed.

Musical stimuli provide participants with ample opportunity to gather information about objective stimulus quality, since the presented musical excerpts provide a constant and relatively long stream of (new) information compared to other sensory stimuli. The long duration should allow participants to gather enough information to form an objective judgment rather than a context-dependent one. By capitalizing on the relatively long duration of musical stimuli, we were able to disentangle cognitive processes related to the behavioral bias formation from processes that were necessary to reduce this potential bias.

## Method

### Participants and Stimuli

**Figure 1:**
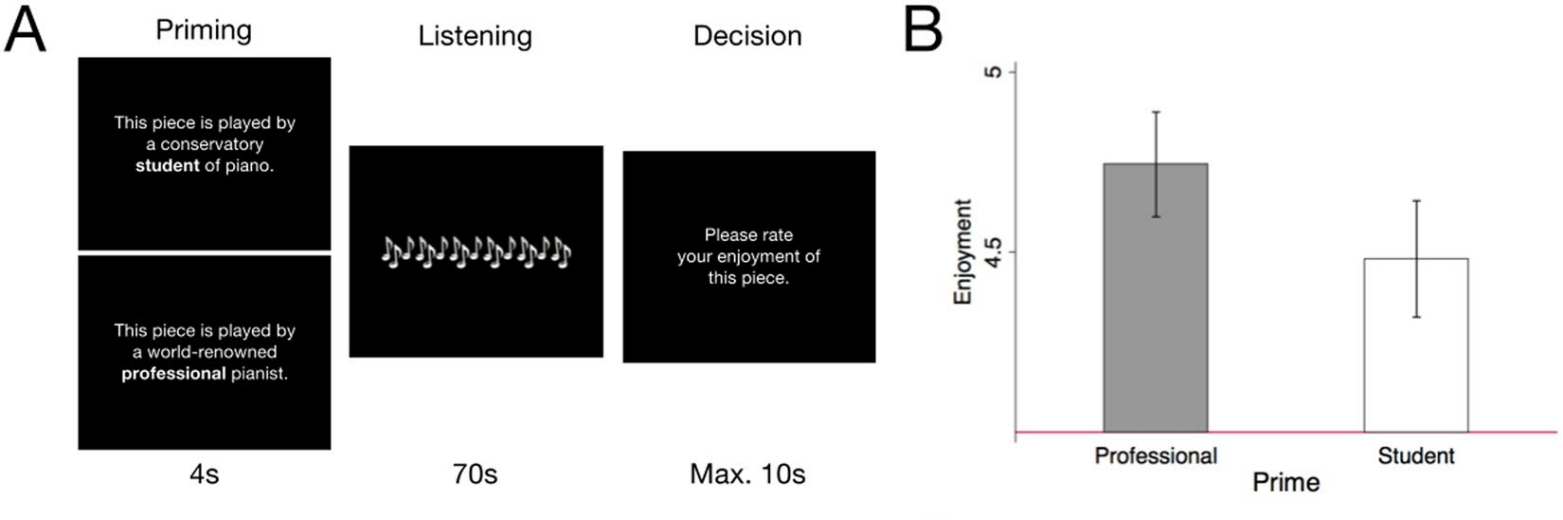
(**A**) Participants listened to pairs of performances of musical excerpts. For each pair, they were told that one was played by a professional and one by a student. They rated ‘enjoyment’ on a 1–7 scale. (**B**) Trial instructions biased enjoyment ratings (Wilcoxon signed-rank test, *z* = 2.750, *p* = 0.006, paired two-sided test).

Twenty participants without formal training in music volunteered to take part in the study (11 female; mean age 24.4 ± 10.3 years). Participants heard eight pairs of 70 s excerpts from piano performances in the Western classical tradition (details of which are listed in Supplementary Information S1). Each pair consisted of two different performances of the same excerpt. Instructions informed participants that one was performed by a “conservatory student of piano” and one by a “world-renowned professional pianist.” Each pair was heard twice at different times during the experiment, once with the first performance primed as professional and the second primed as student, and once with these labels reversed. Framing conditions were counterbalanced, and the trial order was randomized. Since the actual excerpts and the framing conditions were perfectly counterbalanced, any behavioral or neural differences should arise from the framing stimulus and not from the actual quality of the performance. Figure 1 shows how each trial proceeded. First, a professional or student description was displayed for 4 seconds (the framing period), after which the musical excerpt followed for 70 seconds. Participants were then given 10 seconds to rate their enjoyment of the piece on a 1–7 Likert-like scale. The other frame was then displayed for 4 seconds, and the other performance of the musical excerpt was played for 70 seconds followed by the second enjoyment question. Finally, participants were given 10 seconds to indicate which of the two performances they preferred. Presenting participants with the same audio following both frame pairs ensured that all behavioral and neural differences arose exclusively from the expectations of participants, as opposed to the excerpts themselves.

### Procedure

All participants provided written informed consent, were screened for scanning, and were debriefed at the conclusion of the study. Experimental protocols were approved by the University of Connecticut Institutional Review Board, and the methods were carried out in accordance with all relevant guidelines and regulations. We focused our analysis on activity in areas sensitive to auditory and reward-related stimuli and cognitive control. Following existing literature on these areas, we focused on functionally-defined regions within the dlPFC (a critical node in the fronto-parietal control network^16–21^), the primary auditory cortex (Heschl’s region), and the vmPFC (a critical node in brain reward network that responds to primary^18^ and monetary rewards^11,22^).

We analyzed data from all 20 participants. Based on a previous behavioral study^5^, we chose this sample size to ensure capacity to detect a behavioral effect of professionalism on pleasure ratings. This sample size matches the one typically used in imaging studies about framing effects, and is considered to provide sufficient power to detect framing effects in other domains^1,2,10^.

### Imaging, fMRI analysis and DTI analysis

MR data were collected using a Siemens Prisma 3 T scanner with a 20 channel coil. We collected T2-weighted functional images using an EPI sequence (TR = 2 s, TE = 30 ms, 32 axial slices with a thickness of 3 mm and additional spacing of 0.72 mm between the slices), with a voxel size of 2 × 2 × 3 mm and an acquisition matrix of 96 × 96. In each scan, we collected 1455 volumes, but the first volumes were discarded due to tissue saturation effects. High resolution T1-weighted anatomical scans were also collected using an MPRAGE sequence (TR = 2300 ms, TE = 2.28 ms, flip angle = 7°, Image dimensions 256 × 256 × 192, with a slice thickness of 1 mm and a voxel size of 1 × 1 × 1 mm). Diffusion weighted images (used to estimate diffusion tensor images, DTI) were collected using a time echo (TE) of 69 ms, a time repetition (TR) of 6100 ms and 36-diffusion directions defined evenly across the sphere. To perform distortion correction, we collected two images in opposite encoding directions (AP and PA), both collected with a b-value of 1000 s/mm^2^ and a voxel size of 2.0 × 2.0 × 2.5 mm.

Functional imaging analysis was performed using FSL^23^, whereas single-subject analysis was carried out with FEAT (fMRI Expert Analysis Tool) version 6.00. Images were high-pass filtered with a cutoff of 100 s and were smoothed with a 4 mm full-width at half maximum (FWHM) Gaussian kernel. To identify significant neural activity, we thresholded images using clusters determined by Z > 2.3 and a corrected cluster significance threshold at *P* = 0.05. Moreover, we used two methods to analyze the fMRI data on a single-subject level. To examine the evolution of neural activity over the duration of both the framing and listening periods (for a total of 74 seconds), we estimated each subject’s neural activity using a finite impulse response (FIR) model with interval lengths of 2 TRs (i.e. 4 seconds). We used a resolution of 4 seconds to limit the number of regressors needed to estimate the model, since a higher temporal resolution (i.e. 2 seconds) caused problems of multicollinearity with regressors close to linear dependency. Additionally, to get robust estimates for the whole listening period and to disentangle activity caused by the framing stimulus and the music stimulus, we analyzed the data based on a event-related GLM with a double-gamma HRF convolution.

To obtain average activity across subjects, a fixed effects model was estimated with FLAME (FMRIB’s Local Analysis of Mixed Effects) that forced the random effects variance to zero^24^.

The pre-processing of the diffusion images included eddy current correction and Bayesian estimation of diffusion parameters obtained using sampling techniques with modelling of crossing fibers (bedpostx) in FSL. Both the Bayesian estimation of diffusion parameters of a two fiber model and the probabilistic tractography estimation was performed on a GPU^25^. Dual-fiber models obtain more reliable results compared to single-fiber models, since they account for crossing fibers. All analyses were carried out in individual diffusion space after transforming ROIs into that space for each individual. We visually inspected all images to ensure accurate registration and transformation. To obtain measures of white-matter connectivity that correspond to known fiber tracts, we only included voxels in seed space that most likely projected to the target ROIs. We thresholded the obtained probabilistic tractography maps of all non-zero voxels in a seed ROI at 90%, and then computed the median of all remaining voxels in seed space. We used this value as the estimate of the white-matter connectivity between seed and target ROIs^26,27^.

## Results

### Behavioral Analysis

To test whether our experimental paradigm was able to generate a measurable behavioral bias, we compared ratings for professionally framed performances with student-framed performances (see Fig. 1B). A nonparametric test revealed a significant difference in subjective value as a function of framing condition (Wilcoxon signed-rank test, *z* = 2.750, *p* = 0.006, paired two-sided test). Participants rated excerpts framed as the performance of a professional (*M* = 4.74, *SD* = 0.65) significantly higher than excerpts framed as the performance of a student (*M* = 4.48, *SD* = 0.72). This confirms that the experimental paradigm was able to produce the predicted behavioral effect, replicating the findings in^5^. Furthermore, to account for possible influencing factors other than the framing, we regressed in a mixed effects model participant’s self-reported musical ratings on the framing condition (professional vs. student), on whether the performer was actually a professional or a student, and on the order of the presented frame. In keeping with reported results, only the framing condition exhibits significance (*P* = 0.006), whereas participants were not able to ‘guess’ the actual performer (*P* = 0.29). The presented order of the frame exhibits a trend (*P* = 0.058) (see full regression table in Supplementary Table 2).

### fMRI Analysis

#### Differential neural activity related to the enjoyment of a musical excerpt

To better understand the cognitive processes that led to a positive bias in favor of the professional, we analyzed neural activity that preceded a preference for excerpts labeled as played by a professional player compared to the neural activity that preceded a preference for excerpts labeled as played by a student player. Thus, we first computed a whole brain contrast between trials in which a professionally framed performance was preferred with trials when a student-framed performance was preferred. This enabled us to detect any differences in the evidence accumulation phase (i.e. the listening period) across conditions and to shed light on the underlying neural mechanisms leading to the observed disparity in preferences.

**Figure 2:**
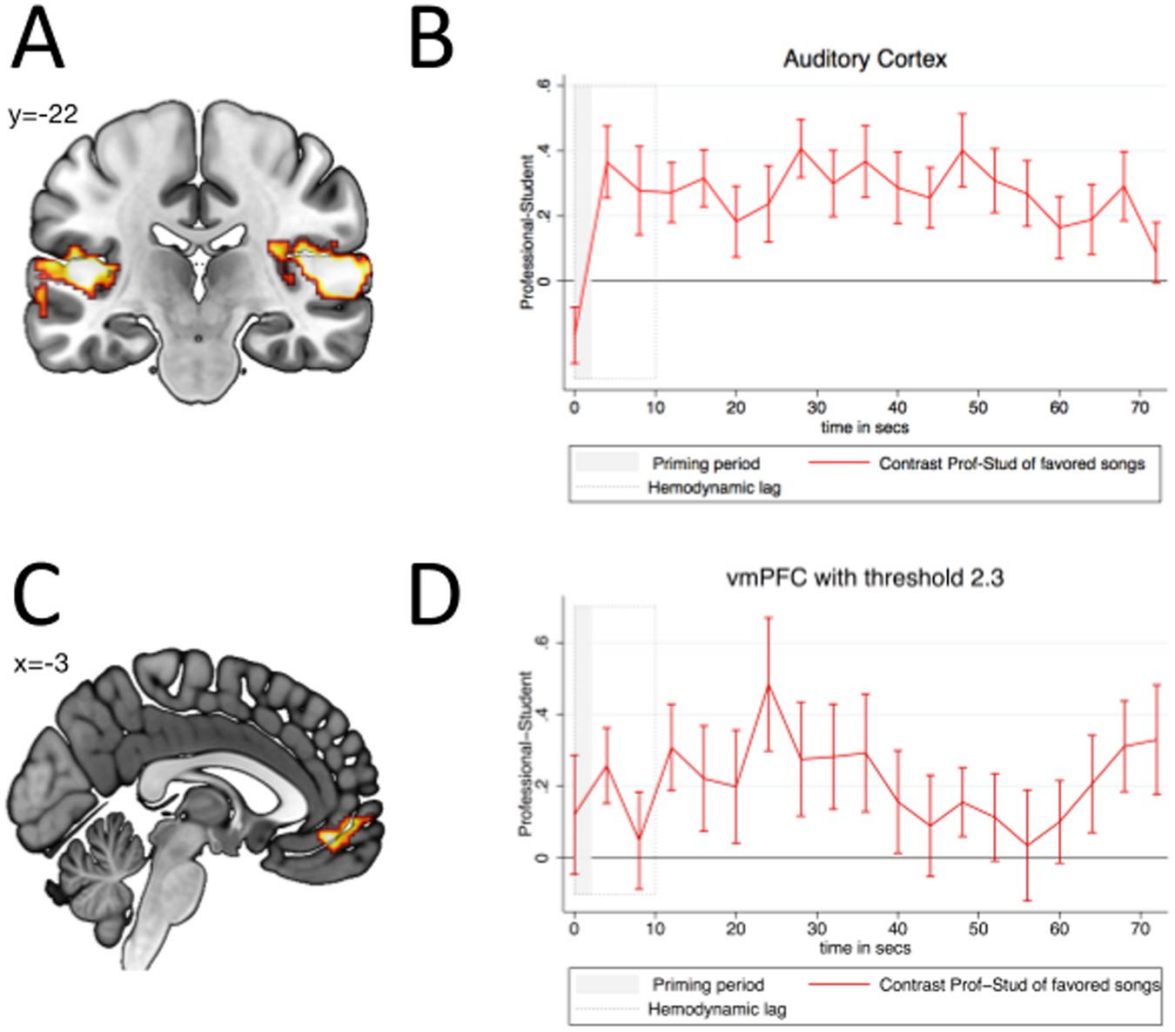
We computed a whole brain contrast between trials in which a professionally framed performance was preferred and trials when a student-framed performance was preferred. (**A**,**B**) Provided an excerpt was preferred, the professional pianist frame induced significantly more activity in the primary auditory cortex relative to the student pianist frame. (**C**,**D**) Greater activation was also found in the vmPFC following the professional frame relative to the student frame.

Our analysis revealed that, when a piece was preferred, the professional pianist frame induced significantly more activity in the primary auditory cortex relative to the student pianist frame (see Fig. 2A). This suggests that beliefs regarding the quality of a performer engendered a bias in attention.

To better understand the evolution of activation during the information accumulation phase in the primary auditory cortex, we extracted brain activity that correlated with the framing stimulus over both the framing and listening periods (see Fig. 2B). We observed higher activation in the primary auditory cortex when the player was described as a professional pianist relative to when the player was described as a student. Moreover, this difference in activity remained consistent, exhibiting no significant changes across the 70 seconds of the excerpt. A panel regression of activity in the primary auditory cortex on time showed no significant linear slope (*b*_1_ = 0.0003, *z* = 0.56, *p* > 0.5). This supports the notion that a bias in attention began almost immediately (i.e. 4 sec) after the presentation of the framing information and remained stable throughout the evidence accumulation period. Contrary to the notion that more evidence should diminish any framing effects generating during the relatively short framing period (i.e. 4 sec), we found that the professional framing gave rise to a constant *attentional* bias in favor of the professional player.

To address the question of how this *attentional* bias materializes in the *behavioral* tendency to prefer professionally framed performances, we examined activity in the vmPFC – a region repeatedly shown to play a critical role in the evaluation and encoding of primary and monetary rewards. To identify the region of vmPFC relevant to our task, we tested for brain regions that were more active following professional framing relative to student framing. This analysis identified the region of the vmPFC shown in Fig. 2C and D. Similarly, in averaging over all time points during the framing and listening periods, we found that an overlapping region of the vmPFC signal was significantly biased in favor of the professionally framed performance (Wilcoxon signed-rank test, *z* = 2.203, *p* = 0.0276, two-sided). We also tested whether framing-related differences in this regions’ activity changed across time as participants gained more direct experience with the musical piece. A panel regression found no significant linear slope (*b*_1_ = −0.0002, *z* = −0.14, *p* > 0.5). Taken together, these findings suggest the presence of a confirmation bias, with the same musical excerpt attracting more attention and correspondingly increased neural signal in the auditory cortex, accompanied by an increase of the neural signal related to subjective value (i.e. vmPFC) when the player was viewed as an established professional in comparison to a conservatory student. Brain activity during the framing period further supports this notion (see Supplementary Information Fig. S1). Particularly, activity in the vmPFC during the framing period—before a single note had been played—correlated with the extent of the behavioral bias. This indicates that the bias was activated in response to the information frame and persisted throughout the duration of the performance (see also Supplementary Information Fig. S2).

#### Neural activity related to overcoming professional-student bias

The question remains: how did some participants, at least during some trials, successfully overcome the professional-vs.-student bias and express a preference for the student performance? Two possibilities exist. In behavioral analyses, biases are assumed to produce a response tendency, with variability around this tendency conceptualized as a nuisance noise term. If positive responses to excerpts primed as played by a student merely represented a kind of error or noise, trials in which the performance was primed as student should be associated with no consistent pattern of brain responses. Conversely, it is also possible that there is something systematic about these responses, with the response bias acting as an initial response tendency that must be explicitly overcome. If this were the case, we would expect that regions of the executive control network associated with response inhibition or cognitive control to consistently be activated during trials when the student performance was reported as preferred.

To distinguish between these possibilities, we computed a contrast between activity during unfavored performances that were framed as played by a professional and those that were framed as played by a student. In both of these cases, participants reported the same low preference. Differences in neural activity may therefore be attributed to differences in the processing of the information provided prior to the listening period. That is, by computing the difference between trials framed as professional and those framed as student in cases where the trials were not preferred, we were able to analyze the cognitive processes involved in overcoming the framing effect and resisting the tendency to prefer the professional.

**Figure 3:**
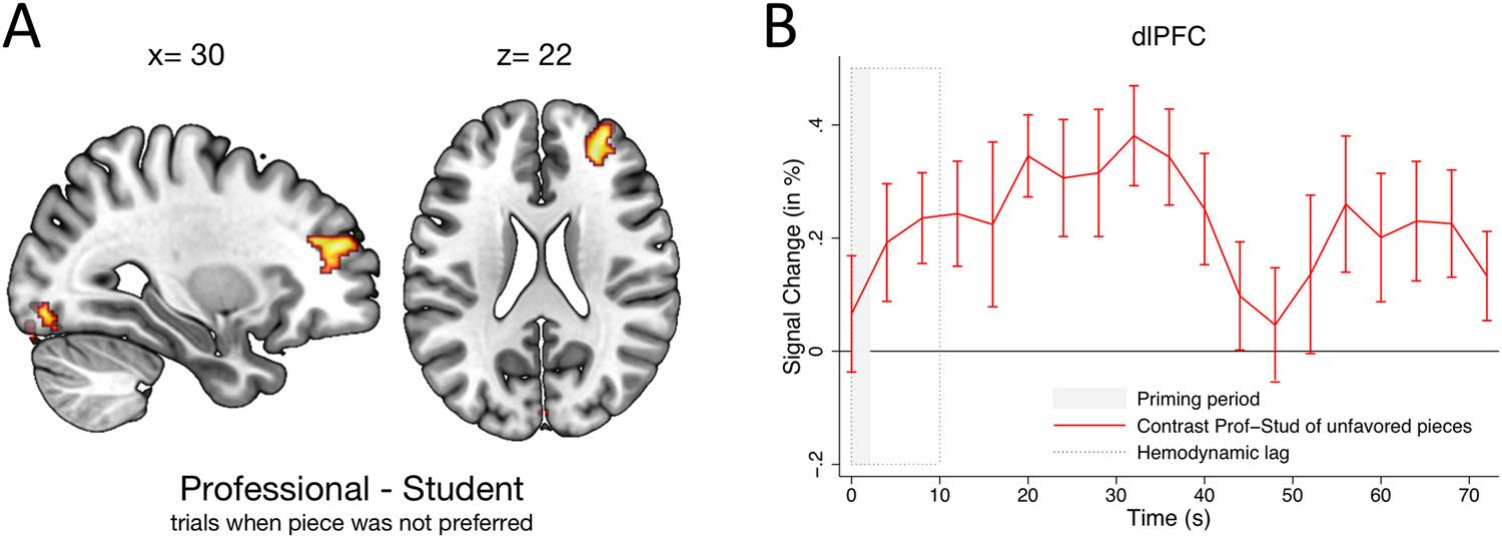
(**A**,**B**) We computed a whole brain contrast between trials in which a professionally framed performance was *not* preferred and trials when a student-framed performance was *not* preferred. This contrast identified a region of the dlPFC, indicating that dlPFC activity correlated with instances when the professionally framed performance was perceived as less impressive compared to cases when the student-framed performance was not preferred.

As shown in Fig. 3A, relative to student-framed performances that were not preferred, professional-framed performances that were not preferred elicited higher activity in the dlPFC, a region related to cognitive control and deliberative effortful thinking processes^16–21^. The development of neural activity in the dlPFC (see Fig. 3B) remained consistently biased toward the professional throughout the listening period (Wilcoxon signed-rank test, *z* = 3.472, *p* = 0.0005, two-sided). This suggests that, as information about the quality of the performance accumulated, participants needed to exert cognitive control in order to form and retain a negative evaluation for performances that had been framed as played by a professional compared to those that had been framed as played by a student. These data suggest that less cognitive effort was required to dislike a performance when it had been described as played by a student rather than a professional. Once again, this pattern of neural activity is consistent with what would be expected from mechanisms underlying a confirmation bias. According to this reading, a low quality auditory experience is easier to associate with a student than a professional. The dlPFC – a critical part of the executive control network – activates differentially when deliberatively overcoming the initial bias evoked by the frame of professionalism.

Based on these findings, we assumed that activity in both regions–the vmPFC and dlPFC–would also be indicative of the *magnitude* of the behavioral bias shown by participants, extending beyond a simple dichotomy of expressed preferences. To confirm that activity in these two regions predicted the magnitude of the actual behavioral bias, we first extracted the activity for each subject in the vmPFC, the dlPFC, and two additional control regions that also showed a significant difference in activity across framing conditions (in fusiform gyrus and left orbitofrontal cortex, OFC). We then regressed the magnitude of the behavioral bias on the extracted activity of each identified region that was averaged across all voxels in a simple OLS regression (see Table 1). Our analysis reveals that only neural activity in the vmPFC had a significant positive impact on the magnitude of the behavioral bias, whereas regions in the sensory (fusiform) and lateral OFC were not correlated with the size of the behavioral bias. Additionally, dlPFC activation correlated with a significantly reduced behavioral bias, suggesting that the exertion of cognitive control counteracts the behavioral bias induced in the vmPFC.

**Table 1:** Predicting the Magnitude of the Behavioral Bias.

Predictor	Magnitude of Behavioral Bias (R^2^ = 0.426)
Fusiform Activity	0.295 (0.158)
Left lateral OFC Activity	−0.208 (0.637)
**vmPFC Activity**	**0**.838* (**0**.**300)**
**dlPFC Activity**	**−0**.788* (**0**.**372)**
Constant	0.022 (0.137)

Note: The table reports unstandardized regression coefficients, with standard errors clustered on participant level in parentheses (*N* = 20). Activity was extracted and averaged across all voxels for each ROI that exhibited significant activity in the contrast depicted in Fig. 2. The behavioral bias exhibited by subject *i* was defined as the difference of the average ratings between performances framed as professional and as student. *p < 0.05. **p < 0.01.

We conclude that the consistent bias in vmPFC activity indicated a preference in favor of the ostensibly professional pianist over the ostensibly student one, and additionally, that the magnitude of this behavioral bias is mitigated by the exertion of cognitive control in the form of dlPFC activation.

#### White-matter structure of cortico-striatal fiber tracts predict framing effects

Given the relationship between trial-level dlPFC activity and behavioral bias, we reasoned that individual differences in the connectivity between the executive control network and areas of the reward network would predict participants’ behavioral bias. Two methods are available to test this prediction. First, diffusion weighted imaging may be used to estimate individual anatomical connectivity of cognitive control structures with the reward network. We predicted that this structural connectivity would negatively correlate with the bias to prefer a professional player. Alternatively, functional connectivity between dlPFC and vmPFC can be assessed using psychophysiological interaction (PPI) analyses^18^. DTI and PPI analyses ostensibly both measure brain connectivity. For brevity, we report DTI analyses below and refer readers to Supplementary Information (S4) for the qualitatively equivalent results using PPI.

**Figure 4:**
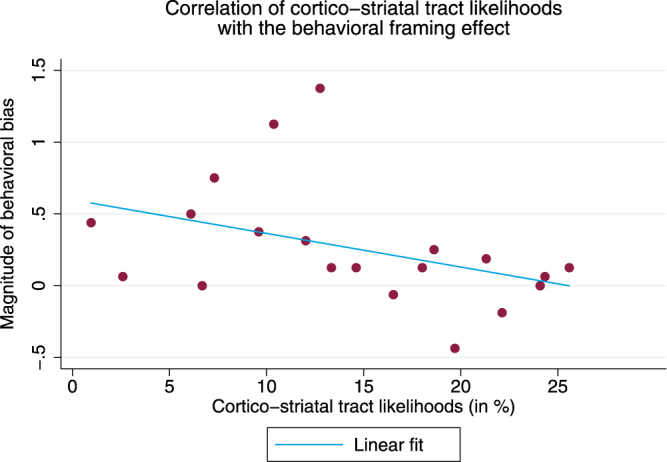
The results of probabilistic tractography were plotted against the magnitude of the behavioral framing effect. Specifically, based on our results from the fMRI analysis, we computed the likelihoods of two cortico-striatal tracts, the dlPFC-caudate and vmPFC-caudate, separately for each participant. We then plotted the averaged cortico-striatal tract likelihoods against the magnitude of the behavioral bias. A non-parametric test revealed a significant correlation between the magnitude of the framing effect and the averaged likelihood of both cortico-striatal tracts (Spearman’s rho = −0.4917, P = 0.0277, two-sided).

We based our analysis strategy on previous findings demonstrating the importance of striatal structures in integrating and interfacing input from different networks with each other^15^. In keeping with neuroanatomic findings regarding the integration of separate cortical loops in the striatum^14,15^, we focused our analysis on the structural connectivity of both pathways, from the dlPFC to the striatum, and from the vmPFC to the striatum. In particular, the caudate is known to receive afferent projections from across the frontal cortex, with separable patches associated with dlPFC and vmPFC connectivity^14^. Based on the specific properties of the caudate within the cortico-basal ganglia circuit and its role in connecting important parts of the cognitive control network with reward structures, we conducted probabilistic tractography with the dlPFC activation mask as a seed and the caudate as a target region. Analogously, we then performed the same probabilistic tractography with the vmPFC activation mask as a seed and the caudate as a target region (see Fig. 4).

To obtain a single measure for structural connectivity of cortico-striatal fiber tracts that link and integrate reward structures with cognitive control structures, we computed the average likelihood of both fiber tracts connecting to striatal regions. Since possible fiber tracts need to be restricted to the originating hemisphere, and task-related dlPFC activation was only present in the right hemisphere, we used the right caudate as the nexus between the dlPFC and the vmPFC (see Supplementary Information Fig. S4). An ipsilateral caudate mask obtained qualitatively similar results.

In line with our hypothesis, we found that the average connectivity of both pathways (i.e. dlPFC to caudate as well as vmPFC to caudate) negatively correlated with the tendency to prefer a professional player (Spearman’s rho = −0.4917, *P* = 0.0277, two-sided). To confirm our results, and to differentiate activity-related effects from structural connectivity between these regions, we regressed the behavioral bias on the structural connectivity of both identified cortico-striatal pathways together with activation in vmPFC and dlPFC (see Table 2). Thus, controlling for activation, participants with greater structural connectivity along the sum of both frontal striatal pathways demonstrated a significantly lower extent of the behavioral bias and showed a lower propensity to prefer the professional player.

**Table 2:** Predicting the Magnitude of the Behavioral Bias.

Predictor	Magnitude of Behavioral Bias (R^2^ = 0.420)
vmPFC Activity	0.628* (0.271)
dlPFC Activity	−0.691* (0.275)
**Structural connectivity of cortico-striatal pathways**	−1.793* (0.684)
Constant	0.324* (0.145)

Note: The table reports unstandardized regression coefficients, with robust standard errors. Activity was extracted and averaged across all voxels for each region that exhibited significant activity in the contrast depicted in Fig. 3. The behavioral bias exhibited by subject *i* was defined as the difference of the average ratings between performances framed as professional and as student. *p < 0.05. **p < 0.01.

## Discussion

The present work examines the role of cognitive control, as represented by activation and structural integration of the dorsolateral prefrontal cortex, in reducing susceptibility to framing effects while listening to musical excerpts. We replicate past findings regarding the importance of vmPFC activation in the materialization of framing bias. Additionally, we extend those findings by demonstrating the executive control network’s ability, in the form of both (a) task related dlPFC activation and (b) its white-matter structural connectivity to the reward network, to mitigate this behavioral bias.

Despite the fact that perceptual evidence from the piano performance accrued over a relatively long time period (i.e. 70 seconds), participants’ experience was still demonstrably biased by the comparatively brief (4 second) description of the player’s supposed quality. Replicating past results, our data also indicate that vmPFC activation predicts both the experienced subjective value of a musical stimulus^10,11,28^ and the corresponding behavioral bias favoring the professionally framed player^2,3,10^. The vmPFC has been shown not only to underlie subjective experiences of emotional value^29^, but also, more specifically, to underlie valuation that incorporates conceptual information to produce “affective meaning”^30^. Novel to this study is the revelation that this bias can persis across a 70 second duration of sensory input.

Our data also indicate that the dlPFC activation and its connectivity along cortico-striatal pathways with the vmPFC play an important role in overcoming this behavioral bias, and consequently, in diminishing susceptibility to contextual information. Given that framing the excerpt as professional biases listeners to experience it positively, the recruitment of the dlPFC likely signifies that it takes extra cognitive effort, and the intervention of executive control, to suppress this bias and instead find the professionally framed performance less impressive. This is in line with research demonstrating selective dlPFC activation when a person resists temptation^31^, exerts self-control in food choice^9^, and overrides negative race-based responses in an implicit association task^32,33^. The necessity of recruiting executive control areas to suppress pre-potent inclinations supports the notion that the bias had already emerged during the framing stage, before any perceptual evidence occurred. Additional data, gleaned from analyzing brain activity during the framing period, further supports this hypothesis (see Supplementary Information Fig. S1). Activity in the vmPFC during the framing period—before a single note had been played—correlates with the extent of the bias it elicited, as measured by behavioral responses collected after the 70-second listening period. Thus, the bias was activated in response to the information frame and persisted throughout the duration of the performance; it did not emerge gradually during the listening period.

Furthermore, based on neural activation over time, we argue that the observed auditory cortex activation likely indicates an increase in attention directed to professionally framed performances^34^. Combined with higher vmPFC activation and correspondingly higher subjective value^11,28^, we argue that closer attention to positive value stimuli resembles a form of confirmation bias^35^. That is, by expecting better performance from a professional, participants directed more attention toward professionally framed pianists compared to the student-framed performances, and therefore, exhibited a heightened tendency to gather more evidence that would confirm their prior expectation about the professional player’s performance.

From the perspective of music psychology, these findings reinforce the notion that extrinsic factors—outside the borders of the notes themselves—can impact perception and evaluation as critically as the intrinsic characteristics of the acoustic signal. Contextual factors known to shape how music is heard include accompanying visual information^36–38^, verbal information such as program notes^5,39,40^, and movement^41^. In addition to providing further evidence for these effects, this study establishes a potential mechanism and timeline for them. Although it uses framing information about the professional status of the performer as the factor of interest, this manipulation can be understood to serve as a proxy for other real-world kinds of contextual information—for example, the effect of hearing a performance in a beautiful concert hall, or knowing about the renown of a performer. Our study suggests that this contextual information can activate an attentional filter to allow in perceptual information that reinforces the expectation. People may think they’re responding to the notes themselves, but their perceptions and judgments in fact reveal the influence of the context surrounding the sounds.

It is important to note, however, that participants in this study lacked formal training in music. People with more extensive knowledge of a domain might not be susceptible to the same contextual biasing effects, and might even be able to overcome them given sensory information of sufficient duration. A follow-up study using highly trained pianists as participants could illuminate whether these biases extend to people with expertise in the domain under consideration. In a similar vein, an alteration of the used musical stimulus might also influence the observed effects. In particular, familiarity with the excerpts could be an important moderating variable that needs to be addressed by future studies. For instance, instead of using existing musical pieces, future studies could test framing effects with improvised or self-composed musical excerpts^42^.

Our data also indicate that besides the importance of dlPFC activation in reducing susceptibility to framing effects, the white-matter structure interfacing the cognitive control network with reward areas also plays an important role. We provide converging evidence that the structural connectivity of white-matter tracts linking dlPFC to vmPFC via the striatum influences resistance to framing effects. Specifically, in line with neuroanatomic findings regarding the integration of separate cortical loops in the striatum^15^, we find that the structural connectivity of the full pathway from the dlPFC to the striatum, and from vmPFC to the striatum negatively correlates with the tendency to prefer a professional player. The caudate in particular has been shown to possess overlapping patches of dlPFC and vmPFC loop-associated connections^14^, and therefore constitutes the most likely nexus between cognitive control and reward areas.

Using functional connectivity analysis (i.e. psychophysiological interactions),^18^ showed that dlPFC activation modulates the value signal in the vmPFC when exerting self-control in a food choice task. We extend their findings by providing initial evidence that this functional connectivity might depend on the actual structure of white-matter tracts connecting and interfacing reward related structures with the cognitive control network. Future research should investigate whether this finding generalizes across different types of cognitive biases and different self-control related tasks, and determine whether striatal structures merely interface or actually integrate the cortical input from different cortical networks while making decisions related to self-control. Future research should also investigate how these processes relate to behavioral outcomes.

Our study provides new insight into the neural circuitry underlying a phenomenon that has long been articulated by philosophers and demonstrated in behavioral studies: the activation of a cultural category—such as a professional or student performer—can affect the perception and valuation of a stimulus, even over a relatively long duration. We extend those findings with initial evidence suggesting that deliberative thinking can mitigate the behavioral bias that arises from affective responses. Accordingly, our findings are relevant for behavioral economists, psychologists and artists alike, as they demonstrate that ‘deliberative and effortful thinking’ can play a crucial role in overcoming cognitive heuristics related to socially constructed concepts and stereotypes.

## Electronic supplementary material

Supplementary information
