# Author Correction: Overcoming Bias: Cognitive Control Reduces Susceptibility to Framing Effects in Evaluating Musical Performance

**DOI:** 10.1038/s41598-018-26663-3

**Published:** 2018-05-31

**Authors:** Gökhan Aydogan, Nicole Flaig, Srekar N. Ravi, Edward W. Large, Samuel M. McClure, Elizabeth Hellmuth Margulis

**Affiliations:** 1 Department of Psychology, Arizona State University, Tempe, Arizona USA; 2 Department of Psychological Sciences, University of Connecticut, Storrs, Connecticut USA; 3 Department of Music, University of Arkansas, Fayetteville, Arkansas USA

Correction to: *Scientific Reports* 10.1038/s41598-018-24528-3, published online 18 April 2018

In Figure 2b and 2d, there are errors in the labelling of the figures. The Correct Figure 2 appears below as Figure 1.

**Figure 1:**
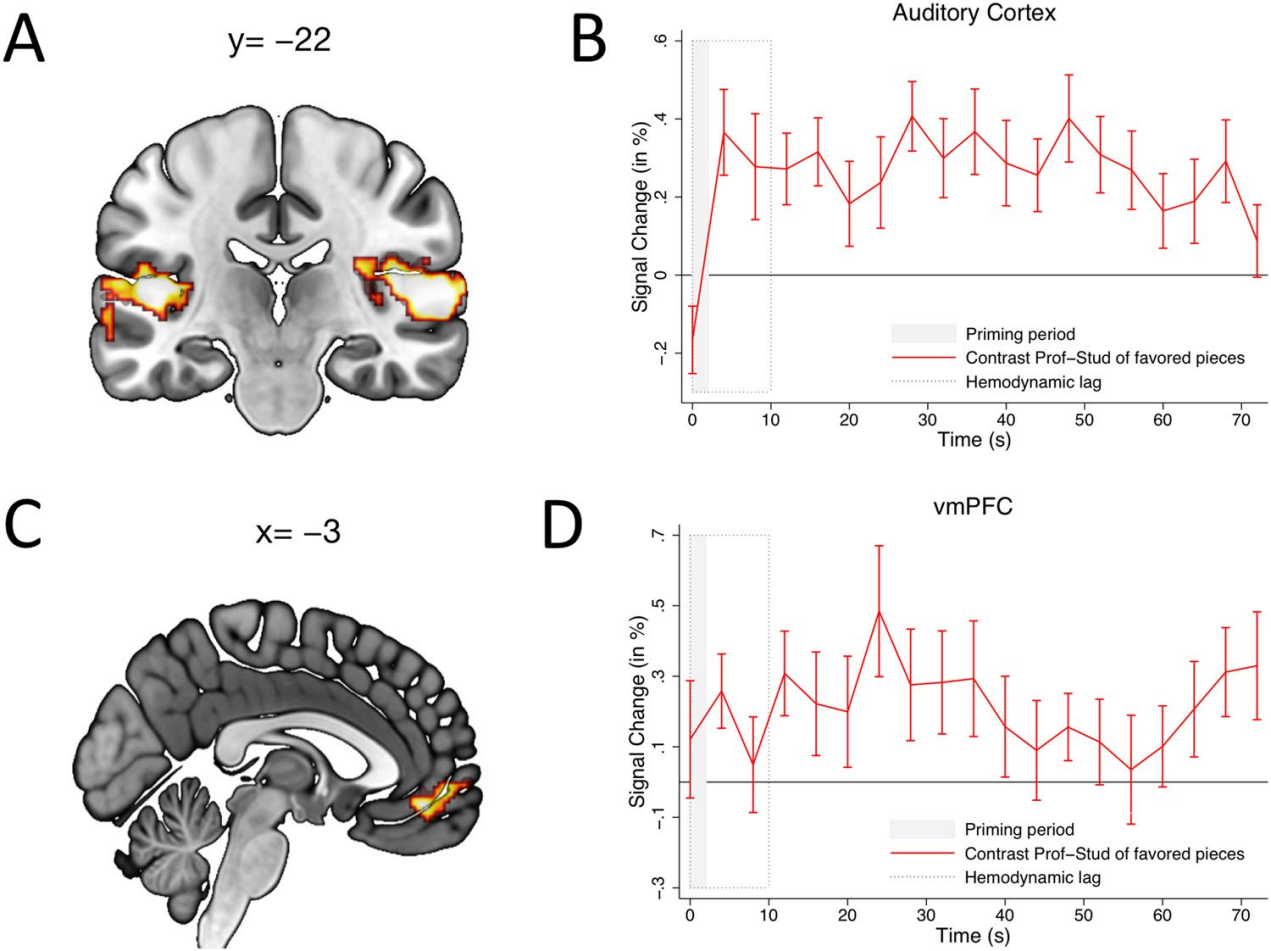
We computed a whole brain contrast between trials in which a professionally framed performance was preferred and trials when a student-framed performance was preferred. (**A**,**B**) Provided an excerpt was preferred, the professional pianist frame induced significantly more activity in the primary auditory cortex relative to the student pianist frame. (**C**,**D**) Greater activation was also found in the vmPFC following the professional frame relative to the student frame.

