# Supplementary material for: Overcoming Bias: Cognitive Control Reduces Susceptibility to Framing Effects in Evaluating Musical Performance
Source: Sci Rep. 2018 Apr 18;8:6229. doi: 10.1038/s41598-018-24528-3 (PMC5906609; doi:10.1038/s41598-018-24528-3)
Supplement: Supplementary file 1 — Supplementary information [file 41598_2018_24528_MOESM1_ESM.pdf]

Overcoming Bias: Cognitive Control Reduces Susceptibility to Framing Effects in Evaluating Musical Performance

Gökhan Aydogan[1], Nicole Flaig[2], Srekar N. Ravi[1], Edward W. Large[2], Samuel M. McClure[1], Elizabeth Hellmuth Margulis[3,*]

[1] Department of Psychology, Arizona State University
[2] Department of Psychological Sciences, University of Connecticut
[3] Department of Music, University of Arkansas

Correspondence regarding this article should be addressed to: Elizabeth Hellmuth Margulis, 201 MUSC, University of Arkansas, Fayetteville, AR 72701
ehm@uark.edu

Appendix

Supplementary Information S1

Stimuli

Piece Number	Piece Name	Composer	Professional Performer	Student Performer
1	Piano Sonata in E, Op. 109, II	L.V. Beethoven	Alfred Brendel	Student 1
2	Piano Sonata in B Flat, K. 570, II	W.A. Mozart	Mitsuko Uchida	Student 2
3	Intermezzo in E, Op. 116, No. 4	J. Brahms	Julius Katchen	Student 3
4	Sonata No. 2 in B Flat Minor, Op.35, I	F. Chopin	Leif Ove Andsnes	Student 4
5	Prelude in B Minor No. 10, Op. 32	S. Rachmaninoff	Vladimir Ashkenazy	Student 5
6	Paganini Variations	F. Say	Fazil Say	Student 6
7	From a Log Cabin Op. 62, No. 9	E. Macdowell	James Barbagallo	Student 7
8	Sonata in G Minor, K. 426	D. Scarlatti	Nikolai Demidenko	Student 8

Supplementary Information S2

Supplementary Table 1: Mixed Effects Estimation of Music Rating

Predictor	Musical Rating (N = 640)
**Musical Framing (Professional vs. Student)**	**.262** (.094)**
Order of Framing (Professional first vs. second)	.3 (.158)
Actual Performer (Professional vs. Student)	.087 (.082)
Constant	3.987** (.368)

Note: The table reports unstandardized regression coefficients of a mixed effects model, with robust clustered standard errors on participant level (N = 20). Participant's self-reported musical ratings were regressed on the framing condition (Professional vs. Student), on whether the performer was actually a professional, and on the order of the presented frame. $^*p < .05$. $^{**}p < .01$.

Supplementary Information S3

Similarities in value-related activity across task phases – framing, listening and response periods

Previous research indicates that cues that trigger differences in value impact activity in brain reward areas when the cue is presented (e.g. McClure et al., 2004), during consumption of the rewarding stimulus (e.g. Plassmann et al., 2008), and when submitting evaluative judgments (e.g. Kirk et al., 2009). We were interested in whether brain reward areas showed differences in activity based on trial framing (professional vs. student) during all three phases of our experiment. We therefore performed a GLM analysis with separate events for framing instruction presentation, the duration of the listening period, and the duration of the response period.

To test for differences in brain reward activity during the framing period,

we computed contrasts across conditions (student vs. professional) and tested for a correlation with behavioral bias (Fig. S1A). In line with previous results, we find a significant correlation between vmPFC activity in the framing period and the extent of the behavioral bias. This analysis showed that participants with greater vmPFC activity followed by a professional frame were more likely to prefer a professional over a student.

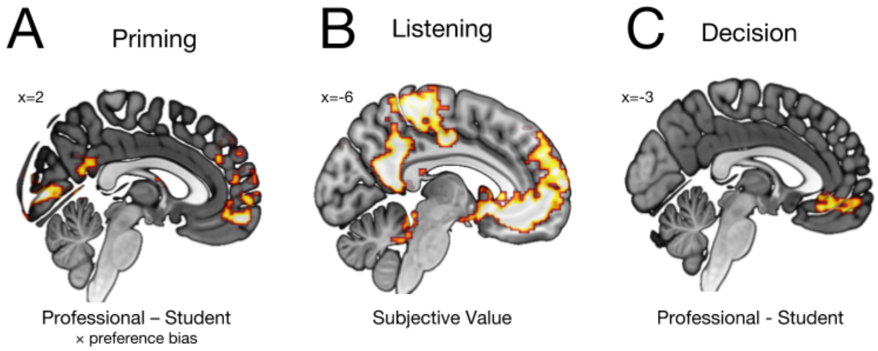

Figure S1. vmPFC activity differed with measures related to subjective value across all task phases. (A) During the framing period, vmPFC activity correlated with the difference in activity across framing conditions (professional – student) to the extent that an overall behavioral bias was observed. (B) As participants were listening to the music, mean vmPFC activity correlated with subsequent ratings of enjoyment. (C) vmPFC activity during the decision period was greater for trials in which instructions indicated that the player was a professional than when it was a student.

By separately analyzing the framing period and the listening period, we are able to extend previous findings by testing whether value expectations persist into the listening period. The previous analysis demonstrated that subjects generate an initial positive subjective value (or positive anticipation) due to

framing. Studying activity during the listening period allowed use to test whether this bias persisted even when the auditory stimulus was identical (across all trials). Analysis of activity during the listening period again indicated a significant correlation between vmPFC activity and subjective value (Fig. S1B). We also observed activity in ventral striatum (VS) and posterior cingulate cortex (PCC) in the listening period; areas that have repeatedly been linked to subjective value in value-related judgments (Bartra, McGuire, & Kable, 2013; Clithero & Rangel, 2014).

Finally, we computed a simple contrast between professionally framed and student-framed trials during the response period (Fig. S1C). We found significant vmPFC activity difference across conditions. In light of the similarities in vmPFC activity during the framing period, the listening period, and the response period, we conclude that the manipulation of expectations (professional vs student performance) lead to a value-related positive anticipation that influenced the perception of the music performance and therefore distorted the final value judgment during all phases of the task.

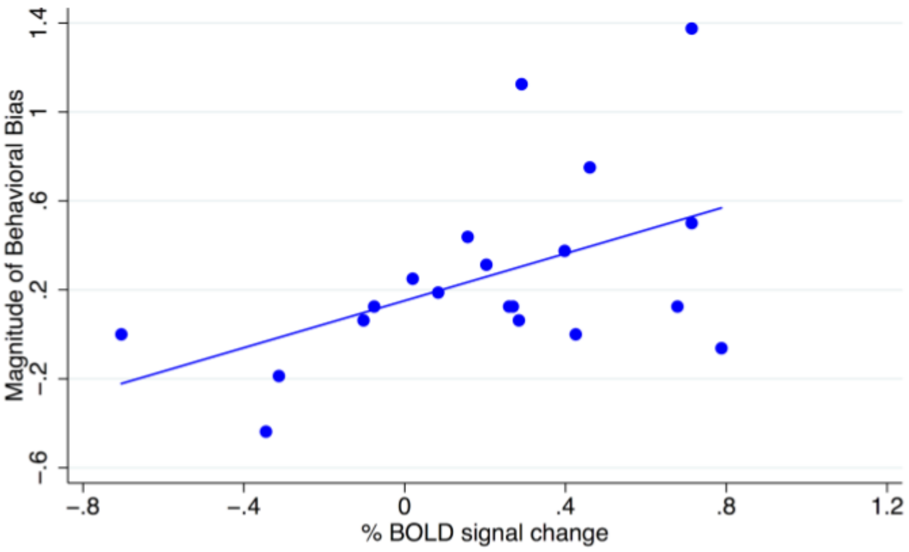

Figure S2. A measure of the behavioral bias is plotted against the difference in BOLD signal change (in %) across framing conditions (i.e. Professional – Student) for vmPFC. Colored lines show the robust linear fit. A higher BOLD signal of professional frames relative to student frames significantly correlates with the extent of the behavioral bias.

Supplementary Information S4

PPI analysis

In the main text, we identified a region of auditory cortex and dlPFC that we argued contribute to music evaluations. We wished to establish whether these cortical regions may feasibly be connected with the vmPFC, where we identified value-related activity. In the main text, we established feasibility by performing DTI analyses to show that the dlPFC and vmPFC are structurally linked via the striatum. Another analytic approach is to test for functional connectivity between regions of interest using psychophysiological interaction (PPI) analyses. We report the results of these analyses here.

First, we investigated whether dlPFC and vmPFC exhibited task related functional connectivity. To this end, we used the dlPFC activation mask from the analysis shown in Fig. 3A. as a seed ROI. Since we were specifically interested in functional connectivity with vmPFC, we used the vmPFC activation mask from Fig. 2C to pre-threshold our results. Consistent with prior research (Hare et al., (2009), we found negative task-related functional connectivity between dlPFC and vmPFC (*P*<.001) during the listening periods of the experiment (Fig. S3A).

To explore the task related functional connectivity of the auditory cortex with reward areas of the brain, we repeated the same analysis, but with the auditory cortex as the seed ROI. Fig. S3B shows the results of the PPI analysis examining task related connectivity of the auditory cortex with vmPFC. Again, we find a significant task related functional connectivity between the auditory cortex and vmPFC (*P*<.01).

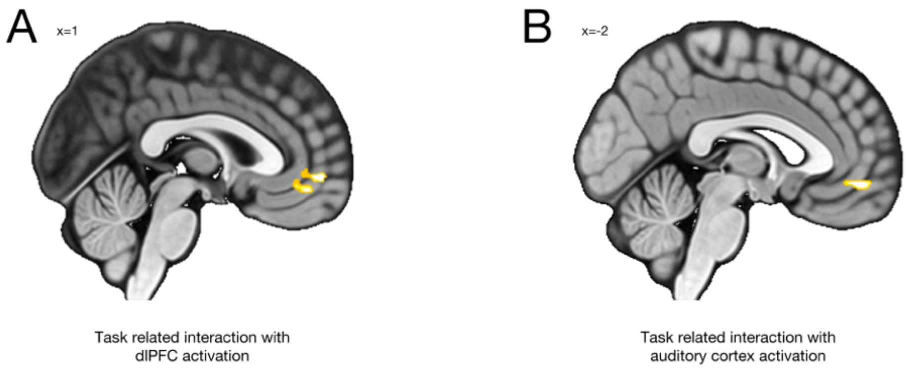

Figure S3. (A) Task related dlPFC activation shows a significant negative functional connectivity to the vmPFC. (B) Analogously, task related auditory cortex activation shows a significant functional connectivity to the vmPFC.

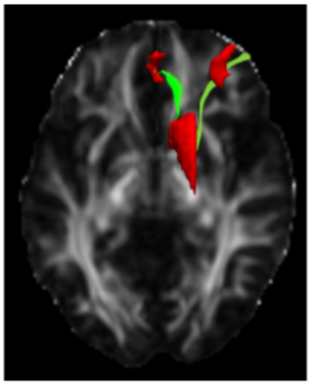

Figure S4. White-matter fiber tract of a typical subject showing tracts connecting the caudate with the dorsolateral and the ventromedial prefrontal cortex.

Supplementary Table 2. Location of activation effects when both the professionally framed or student-framed performance were preferred.

Anatomical Description (Harvard-Oxford Cortical Structural Atlas)	Center of Gravity	Size (mm^3)	Peak Location	Peak Z-Score
Heschl's Gyrus (auditory)	53.3, -13.9, 7.78	37155.36	54, -12, 6	8.84
Heschl's Gyrus (auditory)	-49.7, -18, 6.55	24388.32	-48, -22, 6	6.97
Paracingulate Gyrus	-2.43, 48.6, -8.14	4999.68	-2, 44, -10	4.37
Frontal-medial Orbital Cortex (vmPFC)	-32.8, 33.8, -12	3109.92	-26, 32, -12	4.35
Temporal Fusiform Cortex	-30, -3.56, -28.1	2871.84	-32, -2, -34	3.76

Supplementary Table 3. Location of activation effects when neither the professionally framed or student-framed performance were preferred.

Anatomical Description (Harvard-Oxford Cortical Structural Atlas)	Center of Gravity	Size (mm^3)	Peak Location	Peak Z-Score
Intracalcarine Cortex (visual)	-0.629, -88.8, 1.54	11130.24	-8, -88, 4	5.37
Occipital Pole	19.7, -90, -12	5209.44	16, -98, -10	4.58
Occipital Fusiform Gyrus	-29.4, -77.7, -16.2	3214.08	-32, -76, -16	4.15
Dorsolateral Prefrontal Cortex	30, 47, 20.2	2678.40	34, 56, 20	4.06
Superior Frontal Gyrus, Paracingulate Gyrus	3.85, 15.6, 52.5	2514.72	6, 12, 54	3.58
